# Darwin in Mind: New Opportunities for Evolutionary Psychology

**DOI:** 10.1371/journal.pbio.1001109

**Published:** 2011-07-19

**Authors:** Johan J. Bolhuis, Gillian R. Brown, Robert C. Richardson, Kevin N. Laland

**Affiliations:** 1 Behavioural Biology Group and Helmholtz Institute, Utrecht University, Utrecht, The Netherlands; 2 School of Psychology, University of St Andrews, St Andrews, Scotland, United Kingdom; 3 Department of Philosophy, University of Cincinnati, Cincinnati, Ohio, United States of America; 4 School of Biology, University of St Andrews, St Andrews, Scotland, United Kingdom

## Abstract

Evolutionary Psychology (EP) views the human mind as organized into many modules, each underpinned by psychological adaptations designed to solve problems faced by our Pleistocene ancestors. We argue that the key tenets of the established EP paradigm require modification in the light of recent findings from a number of disciplines, including human genetics, evolutionary biology, cognitive neuroscience, developmental psychology, and paleoecology. For instance, many human genes have been subject to recent selective sweeps; humans play an active, constructive role in co-directing their own development and evolution; and experimental evidence often favours a general process, rather than a modular account, of cognition. A redefined EP could use the theoretical insights of modern evolutionary biology as a rich source of hypotheses concerning the human mind, and could exploit novel methods from a variety of adjacent research fields.

In the century and a half since Charles Darwin's publication of the *Origin of Species*, evolutionary theory has become the bedrock of modern biology; yet, its application to the human mind remains steeped in controversy [1]–[13]. Darwin himself wrote of cognitive evolution, most notably in *The Descent of Man*, where he suggested that like any other trait, human “mental faculties” are the outcome of evolution by natural and sexual selection and insisted that they should be understood in light of what he called “common descent”. This evolutionary interpretation of human cognition was taken up in the 1980s by contemporary evolutionary psychology, which rapidly became dominated by a school of thought stemming from the University of California at Santa Barbara (see Box 1). The essence of this brand of Evolutionary Psychology (EP) is neatly summarized in the famous quote that “Our modern skulls house a Stone Age mind” [2].

**Box 1. The Major Tenets of Evolutionary Psychology**

According to the Santa Barbara school of Evolutionary Psychology (EP), human minds are organized into a large number of evolved psychological mechanisms—psychological adaptations designed to solve recurrent problems faced by our hunter-gatherer ancestors [30]. These evolutionary psychologists attempt to provide criteria for “carving the mind at its natural joints” [104], generally by reverse-engineering from an observable phenomenon to its proposed function.

In the 1980s, four major tenets of EP crystallized, and these ideas became widespread. While not all evolutionary psychologists endorse the Santa Barbara perspective, these ideas have nonetheless shaped the broader field, and remain extremely prevalent.

1. *The environment of evolutionary adaptedness* (*EEA*). This concept refers to the notion that our psychological mechanisms have evolved in response to stable features of ancestral environments [87]. While the EEA has frequently been equated with an African Pleistocene savanna, this version of the concept has been strongly critiqued [66], and the more recent formulation of the EEA concept presents a broader, less specific theoretical landscape of our past lives, based on an abstract statistical composite of all relevant past selective environments [105].

2. *Gradualism.* Evolutionary psychologists argue that minds are built from co-adapted gene complexes that are unable to respond quickly to selection [105],[106]. When combined with the concept of the EEA, gradualism suggests that human beings experience an *adaptive lag* [88], such that evolved psychological mechanisms may not produce adaptive responses in modern human environments that have undergone dramatic recent changes [105].

3. *Massive modularity.* Given that different sets of adaptive problems will have required different computational solutions, the mind is argued to consist predominantly of domain-specific, modular programmes [105]. Whether the mind also contains evolved general-purpose processes remains debated within EP [104].

4. *Universal human nature.* The evolved computational programmes in the human mind are assumed to be responsible for producing a universal (that is, species-typical) human nature [105]. At the same time, different outcomes of these programmes are suggested to be triggered by different environmental or social conditions, leading to the prediction of both universal behavioural outcomes and locally specified adaptive solutions [105].

However, many evolutionarily minded psychologists, evolutionary biologists, and philosophers of science disagree with the theoretical proposals put forward by the Santa Barbara evolutionary psychologists, and the discipline has been the subject of intense debates [1],[3]–[13]. Here, we assess the impact of recent developments in genetics, evolutionary and developmental biology, paleoecology, and cognitive science on EP and then go on to suggest that these developments provide new avenues for research.

## Reassessing the Major Tenets of Evolutionary Psychology

EP is encapsulated by four major tenets (see Box 1) that have generated considerable discussion. Here, we argue that all of these basic assumptions need to be reassessed in the light of contemporary evidence.

### The Environment of Evolutionary Adaptedness and Gradualism

EP argues that that human cognitive processes evolved in response to selection pressures acting in ancestral conditions—in an environment of evolutionary adaptedness (EEA)—and are not necessarily adaptive in a contemporary world that has changed radically in recent millennia. From this vantage point, genetic evolution simply could not keep pace fully with the extraordinary rate at which human technology transformed environments. Tied up with this notion of adaptive lag (or mismatch between our biology and our environment) is an emphasis on evolutionary gradualism: evolutionary change, particularly with respect to complex adaptations in the human mind, is deemed to have occurred slowly; too slowly to have led to significant genetic change in the few hundred generations that have elapsed since the end of the Pleistocene, or even since the spread of modern humans around the world over the last 50,000 years.

Recent developments in human genetics have challenged the concepts of adaptive lag and gradualism. EP originated in the early 1980s, when our knowledge of the human genome was limited and gradualism dominated evolutionary thinking (although biologists' attempts to estimate rates of selection in nature were in full flow in the 1970s [14], leaving the Santa Barbara school's gradualism assumption contentious from the outset). Since then, geneticists have not only mapped the genome, but have devised means for detecting which genes have been subject to recent selection [15]–[19]. There have been substantial human genetic changes in the last 50,000 years, with possibly as much as 10% of human genes affected [19]. Events in the Holocene (the last 10,000 years), particularly the adoption of agriculture, domestication of animals, and the increases in human densities that these practices afforded, were a major source of selection on our species [17]–[22], and possibly accelerated human evolution [20],[22]. Evidence from the human genome strongly suggests that recent human evolution has been affected by responses to features of the environment that were constructed by humans, from culturally facilitated changes in diet, to aspects of modern living that inadvertently promoted the spread of diseases [22],[23]. Genes expressed in the human brain are well-represented in this recent selection [11],[12].

Evolutionary biologists have also measured the rate of response to selection in a wide variety of animals [14],[24], finding that evolutionary change typically occurs much faster than hitherto thought. A recent meta-analysis of 63 studies that measured the strength of natural selection in 62 species, including more than 2,500 estimates of selection, concluded that the median selection gradient (a measure of the rate of change of fitness with trait value) was 0.16, which would cause a quantitative trait to change by one standard deviation in just 25 generations [24]. If humans exhibit equivalent rates, then significant genetic evolution would occur over the course of a few hundred years. While fast evolution is far from inevitable, there is nonetheless strong evidence that it has frequently occurred in humans. EP has yet to come to terms with the possibility of recent, rapid genetic changes with their potential for associated neural rewiring.

Even if we consider the selection pressures that acted on ancestral human populations during the Pleistocene epoch (approximately 1.7 million to 10,000 years ago), the abstract concept of stable selection pressures in the EEA is challenged by recent evidence from paleoecology and paleoanthropology. The Pleistocene was apparently far from stable, not only being variable, but progressively changing in the pattern of variation [25],[26]. The world experienced by members of the genus *Homo* in the early Pleistocene was very different from that experienced in the late Pleistocene, and even early anatomical modern *Homo sapiens* that lived around 150,000 years ago led very different lives from Upper Paleolithic people (40,000 years ago) [27]–[29].

### Universalism

EP has also placed emphasis on the concept of human nature, comprising a species-specific repertoire of universal, evolved psychological mechanisms, from a childhood fear of strangers, to a cheater-detection mechanism, to a preference for specific mate characteristics. This putative universal cognition can be rendered compatible with the observed diversity in human behaviour by recourse to context-dependent strategies. From this perspective, the mind shifts between pre-specified behavioural outputs in response to differential environmental influences [30],[31].

This explanation of human behavioral variation is also contentious [3],[32]–[34]. The notion of universalism has led to the view that undergraduates at Western universities constitute a representative sample of human nature, a view that has been subject to criticism from anthropologists and psychologists [33]–[35]. Moreover, by EP's formulation, all epigenetic and developmental effects simply evoke alternative genetically pre-specified strategies. Recent trends in developmental psychology and neuroscience have instead stressed the malleability of the human brain, emphasizing how experience tunes and regulates synaptic connectivity, neural circuitry and gene expression in the brain, leading to remarkable plasticity in the brain's structural and functional organization [36]. Neuroscientists have been aware since the 1980s that the human brain has too much architectural complexity for it to be plausible that genes specify its wiring in detail [37]; therefore, developmental processes carry much of the burden of establishing neural connections.

In parallel, emerging trends in evolutionary theory, particularly the growth of developmental systems theory, epigenetic inheritance, and niche-construction theory, have placed emphasis on organisms as active constructors of their environments [38]–[40]. The development of an organism, including the characteristics of its brain, involves a complex interaction between genetically inherited information, epigenetic influences, and learning in response to constructed features of the physical and social environment [5],[40]–[45]. From this viewpoint, the human mind does not consist of pre-specified programmes, but is built via a constant interplay between the individual and its environment [45],[46], a point made by developmental psychologist Daniel Lehrman [47] many years ago. By constructing their worlds (for example, by building homes, planting crops, and setting up social institutions), humans co-direct their own development and evolution [22],[39],[48],[49].

The view that a universal genetic programme underpins human cognition is also not fully consistent with current genetic evidence. Humans are less genetically diverse than many species, including other apes [50], largely because human effective population sizes were small until around 70,000 years ago [51],[52]. Nonetheless, there is enough genetic variation to have supported considerable adaptive change in the intervening time, and recent thinking amongst geneticists is that our species' unique reliance on learned behaviour and culture may have relaxed allowable thresholds for large-scale genomic diversity [21],[53]. Human behavioral genetics has also identified genetic variation underlying an extensive list of cognitive and behavioural characteristics [54].

While variation within populations accounts for the bulk of human genetic variation, around 5%–7% of genetic differences can be attributed to variation between populations [55]. Some of the significant genetic differences between human populations have arisen from recent selective events [56],[57]. Gene-culture coevolution may well turn out to be the characteristic pattern of evolutionary change in humans over recent time spans [22],[58] (see Box 2). From this perspective, cultural practices are likely to have influenced selection pressures on the human brain, raising the possibility that genetic variation could lead to biases in the human cognitive processing between, as well as within, populations. In summary, there is no uniform human genetic program.

**Box 2. Gene-Culture Coevolution**

Gene-culture coevolutionary theory explores how genetic and cultural processes interact over evolutionary time [22],[58]. Changes in diet afforded by cultural practices, such as agriculture and the domestication of plants and animals, provide compelling examples of gene-culture coevolution, demonstrating how cultural practices have transformed the selection pressures acting on humans and given rise to some of the genetic differences between human populations. For instance, there is now little doubt that dairy farming created the selective environment that favoured the spread of alleles for adult lactose tolerance [85],[107],[108]. Another example concerns the evolution of the human amylase gene: Perry et al. [109] found that copy number of the salivary amylase gene (*AMY1*) is positively correlated with salivary amylase protein level and that individuals from human populations with high-starch diets have, on average, more *AMY1* copies than those with traditionally low-starch diets. Indeed, the transition to novel food sources with the advent of agriculture and the colonization of new habitats would appear to have been a major source of selection on humans [17],[110], and several genes related to the metabolism of carbohydrates, lipids, and phosphates show signals of recent selection [17]–[19].

More generally, human dispersal and subsequent exposure to novel climates, aggregation and exposure to new pathogens, and farming and exposure to new diets are now widely thought to be the source of selection for the spread of many human alleles [22]. Amongst the overrepresented categories in genome-wide scans of recent selection are numerous alleles expressed in the human nervous system and brain [17]–[19]. This raises the possibility that complex cognition on which culture is reliant (social intelligence, language, and challenges associated with constructing and adapting to new environmental conditions) have driven human brain evolution. Mathematical models exploring how genetic and cultural processes interact provide strong support for the role of gene-culture coevolution in human evolution [92],[111]–[115]. Analyses of these models has often revealed patterns and rates of change that are uncharacteristic of more traditional population genetic theory [92,114–116]. Gene-culture dynamics are typically faster and stronger and operate over a broader range of conditions than conventional evolutionary dynamics [22],[83],[117],[118].

EP's emphasis on a universal human nature has hindered its exploitation of new opportunities to examine human diversity utilizing evolutionary biology. Contemporary evolution theory makes predictions about behavioural variation within and between populations in traits commonly studied by evolutionary psychologists. For example, sex differences in mate preferences constitute a large proportion of EP research and are generally assumed to exhibit universal patterns (e.g., [59],[60]); however, sexual selection theory suggests that a number of factors, such as sex-biased mortality, population density, and variation in mate quality, will affect sex roles (see Box 3). A modern EP would make greater use of the theoretical insights of modern evolutionary biology as a source of testable hypotheses [3],[6].

**Box 3. Reconsidering the Evolution of Sex Roles**

Based on the classic work of Bateman [119] and Trivers [120], EP has predicted sex differences in the relative competiveness and choosiness of men and women when seeking mating partners. Men are generally assumed to have been selected to favour more sexual partners than women and to base their choices on the age, health, and physical attractiveness of prospective partners; in contrast, women are assumed to be more choosy than men and to base their judgements on the willingness of males to invest resources in their offspring [59]. However, contemporary sexual selection theory [121],[122] suggests that a number of factors, such as sex-biased mortality, population density, and variation in mate quality, will affect how competitive and choosy males and females are, with sex roles expected to vary considerably within and between societies. For example, this theory predicts that, in human beings, both sexes will be choosy when encounter rates with potential mates are high, particularly where the parental investment levels of both sexes are large and not too different, and/or where variation in mate quality of both sexes is high, and males are likely to be choosy in populations with a female-biased adult sex ratio and considerable paternal investment.

The prediction that sex roles will vary between populations is borne out in data on variance in mating and reproductive success in current and historic human populations, which does not support the notion of a single universal pattern [123]. In addition, evolutionary psychologists have themselves begun to record cross-cultural variation in mate preferences and to examine whether variables such as adult sex ratios and local pathogen loads can explain within- and between-population variation in mating behaviour (e.g., [31]). However, the EP perspective generally assumes that context-specific strategies are pre-programmed within our evolved psychological mechanisms, such that individuals possess multiple strategies that are differentially elicited by certain external factors or that individuals develop a particular strategy as a result of environmental inputs acting on evolved developmental systems during early life (e.g., [30],[60]. Arguably, the more flexible and variable the exhibited behaviour, the less explanatory power can be attributed to evolved structure in the mind.

An alternative perspective, supported by developmental systems and niche construction theorists (e.g., [38],[39]), posits that the human mind does not consist solely of pre-specified programmes and that brain development is strongly influenced by transmitted culture. One of the key contrasts between this perspective and traditional EP is therefore the role that socially transmitted culture has to play in the development of the brain and behaviour [32]. For illustration, consider how the relatively recent developments of agriculture (niche construction), high-density populations, and the evolution of social stratification (transmitted culture), have dramatically changed the ecological context of human mating decisions from what would have occurred in hunter-gatherer societies. According to the aforementioned theory, the increasing encounter rates that such practices likely afforded should have led to much greater choosiness in both modern men and women compared to their Pleistocene ancestors. Modern evolutionary theory has much to offer evolutionary psychologists who are willing to eschew a focus on universality.

### Massive Modularity

EP has proposed that the mind consists of evolved cognitive modules, a perspective referred to as the massive modularity hypothesis [61],[62]. Massive modularity is a somewhat idiosyncratic interpretation of Fodor's [63] original concept of modularity. Essentially, Fodor suggested that what he called input systems (such as those involved in auditory and visual perception, but also in language) were modular, i.e., operating in relative isolation from each other. Information from these modular systems would be passed on to central systems (involved in problem solving or thought) that themselves were thought not to be modular. EP has extended modularity to involve the whole mind/brain.

The thesis of massive modularity is not supported by the neuroscientific evidence [64]–[67]. Firstly, comparative psychology presents an unassailable case for the existence of domain-general mechanisms. The processes of associative learning are widespread in animals and have general properties that allow animals to learn about the causal relationships among a wide variety of events [68],[69]. For instance, a simple learning theory rule, known as the Rescorla–Wagner rule [70], has proved extraordinarily useful in explaining the results of hundreds of experiments in diverse animals, including foraging in honeybees, avoidance conditioning in goldfish, and inferential reasoning in humans.

Secondly, there is broad involvement of diverse neural structures in many psychological processes, and there is feedback even to the most basic perceptual processing. For instance, the hominid brain has not only witnessed a proportional expansion of the neocortex, but the neocortex has become intricately interconnected and has evolved projections into the medulla and spinal cord [71]. This has allowed humans to learn intricate routines of movement and complex manual tasks, because the Fodorian executive part of the brain can directly monitor the fingers and the feet [71]. The same projections allow exhibit fine control of the tongue, vocal chords, and breathing, without which humans probably could not have learned to speak [71]. After evaluating the evidence and consistent with Fodor's original proposals, Bolhuis and Macphail [64] suggested that there is no evidence for modularity in central systems such as those involved in learning and memory. With regard to cognitive mechanisms, more often than not, data from animal experiments is consistent with a general-process account rather than an interpretation involving adaptively specialized cognitive modules [64],[65],[67],[72].

A large part of EP's emphasis on massive modularity drew from artificial intelligence (AI) research. While the great lesson from AI research of the 1970s was that domain specificity was critical to intelligent behaviour, the lesson of the new millennium is that intelligent agents (such as driverless robotic cars) require integration and decision-making across domains, regularly utilize general-process tools such as Bayesian analysis, stochastic modelling, and optimization, and are responsive to a variety of environmental cues [73]. However, while AI research has shifted away from an emphasis on domain specificity, some evolutionary psychologists continue to argue that selection would have favoured predominantly domain-specific mechanisms (e.g., [74]). In contrast, others have started to present the case for domain-general evolved psychological mechanisms (e.g., [75],[76]), and evidence from developmental psychology suggests that domain-general learning mechanisms frequently build on knowledge acquired through domain-specific perceptual processes and core cognition [44]. Both domain-specific and domain-general mechanisms are compatible with evolutionary theory, and their relative importance in human information processing will only be revealed through careful experimentation, leading to a greater understanding of how the brain works [44].

## Towards a New Science of the Evolution of the Mind

We have reviewed how developments in a number of scientific fields have called into question the key tenets of EP. Fortunately, these developments do not just create problems for EP, but also suggest potential solutions. We argue that the key factor will be the methodological and conceptual integration of EP with adjacent fields.

Traditionally, EP has tested hypotheses using the conventional tools of psychology (questionnaires, computer-based experiments, etc.). Generally these hypotheses have a functional perspective—that is, EP proposes that a particular mechanism functioned to enhance reproductive success in our ancestors. However, Nobel laureate Niko Tinbergen [77] famously proposed that understanding behavior requires comprehension not only of its *function* and *evolution,* but also of its *causation* and *development* [78], and he argued that a complete understanding of behavior involves addressing all four of these questions. These distinctions are relevant because accounts of the evolution of brain and cognition cannot in themselves explain the brain's underlying working mechanisms [1], since these are logically distinct questions. While evolutionary analyses may generate clues as to the mechanisms of human cognition, these are best regarded as hypotheses, not established explanations, that need to be tested empirically [1],[64],[79], and there are instances where such evolutionary hypotheses about mechanisms have had to be rejected [1]. Here, we ask which of Tinbergen's questions is currently addressed in the field of EP and describe how EP could expand its focus to provide a broader and richer understanding of human behaviour.

Evolutionary psychologists commonly seek to study how the human mind works by using knowledge of evolution to formulate, and sometimes test, hypotheses concerning the function of cognitive architecture. While functional or evolutionary considerations cannot be used to test hypotheses about mechanisms, considerations in one domain can generate hypotheses concerning problems in the other domain. For instance, a theory of the evolution of a certain cognitive trait may generate hypotheses as to the mechanisms of that trait. Evolutionary psychologists have conducted hundreds of empirical studies to test the predictions generated by consideration of evolutionary arguments [80]. However, we should be clear that such studies do not test the evolutionary hypotheses themselves, but rather test whether the predictions about the psychological mechanisms have been upheld [6],[81]. For example, the numerous studies supporting the hypothesis that human beings are predisposed to detect cheaters in social situations [74],[82] are consistent with several evolutionary explanations. While the original researchers reasoned that cheater detection has resulted from a selective history of reciprocal altruism [82], alternative evolutionary explanations, for instance that a history of cultural group selection has selected for this trait [83], and non-evolutionary explanations, are also plausible.

The recent trend within the behavioural sciences has been away from confirmation or rejection of a single hypothesis towards the far more powerful simultaneous evaluation of multiple competing statistical models through model selection procedures [84]. A modern EP would, as standard practice, conduct empirical studies designed specifically to test between multiple competing adaptive and non-adaptive explanations [13], and would test the evolutionary historical, as well as the proximate, aspects of its hypotheses. In the following sections, we examine how EP could expand to cover all four of Tinbergen's questions.

i) A modern EP would evaluate the *evolution* of a character by constructing and testing population genetic models, estimating and measuring responses to selection, exploring the covariation of phenotypic traits or genetic variation with putative selective agents, making comparisons across species and seeking correlates to selected traits in the selective environment, and so forth, as do contemporary evolutionary biologists. In addition to these established tools, researchers can also exploit modern comparative statistical methods applied to cultural and behavioural variation [85] and gene-culture coevolutionary theory [22],[58],[83],[86] to reconstruct human evolutionary histories. The function of reliable aspects of human cognition, and of consistent behavioural patterns, can be explored utilizing the same methods. An important point here is that researchers are not restricted to considerations of the *current* function of evolved traits, and well-established methods are available to reconstruct the evolutionary history of human cognition.

ii) With regard to *functional* questions, while EP has stressed the idea that human beings are adapted to past worlds [87], a niche-construction perspective argues that human beings are predicted to build environments to suit their adaptations, and to construct solutions to self-imposed challenges, aided and abetted by the extraordinary level of adaptive plasticity afforded by our capacities for learning and culture [88]. While adaptiveness is far from guaranteed, from this theoretical perspective humans are expected to experience far less adaptive lag than anticipated by EP [88]. If correct, examining the relationship between evolved psychological mechanisms and reproductive success in modern environments will not necessarily be an unproductive task.

Consistent with this hypothesis is the observation that humans have experienced extraordinary levels of population growth, indicative of increments in absolute fitness, in the Holocene whilst exposed to modern, culturally constructed environmental conditions [60]. However, rather than simply pronouncing that human behaviour is, or is not, likely to be adaptive, a modern EP would carry out quantitative analyses across a multitude of behavioural and cognitive traits to measure to what extent, or on what occasions, human behaviour is currently adaptive (e.g., [89]). We anticipate that the formal methods of human behavioural ecology are likely to be productive even in modern societies, in many instances (e.g., [90],[91]). Where the use of optimality models proves unproductive, cultural evolution and gene-culture coevolutionary models could be developed to investigate whether the data conform to equilibria that are not globally optimal (e.g., [92]). Researchers could go on to explore which factors explain this variation, for instance by measuring, among diverse traits and across a broad range of populations, what percentage of the variance in behaviour is explained by local ecology and what percentage is better predicted by cultural history (e.g., [93]).

iii) In order to study the *causal mechanisms* underlying the character, researchers can employ methodologies that are available to modern cognitive psychologists and neuroscientists, such as fMRI and related technology, and take advantage of advances in genetics. While much EP research describes human behaviour in terms of information processing, decision rules and cognition, the psychological adaptations can also be described at the level of the nervous system. Cognitive and behavioural neuroscientists have amassed a huge amount of research on the functioning of the nervous system, including the influence of genes on brain development. However, evolutionary psychologists rarely examine whether their hypotheses regarding evolved psychological mechanisms are supported by what is known about how the brain works. Here the role of evolutionary knowledge is less direct, and again relegated to the generation of novel hypotheses that can be tested using established protocols.

Variation in experimental procedures, patterns of connectivity, differences between individuals, and comparisons across species potentially allows researchers to explore to what extent the circuitry associated with the focal mechanism is human specific, and to identify both the major genes involved and the environmental conditions that regulate their expression. There is evidence that modern neuroscience technologies are starting to be used to test hypotheses generated from evolutionary theory [94]–[97], and some evolutionary psychologists are beginning to present evolutionary accounts of genetic variation underlying traits such as personality [98]–[100]. The aforementioned developments in cognitive neuroscience and genetics open up further opportunities for a broader EP.

iv) As discussed earlier, *development* is an extremely important factor in human cognition, and the human mind is built via a constant interplay between the individual and its environment. Recent work by developmental psychologists demonstrates how it is possible to detect the unlearned roots of cognition, such as deep, explicit conceptual understanding, through careful experimentation on young children [44]. Such experiments also reveal the manner in which culturally and individually variable concepts emerge, through domain-general learning akin to bootstrapping, in response to a culturally constructed, symbolically encoded environments [44]. In principle, all posited evolved psychological mechanisms, from fear of snakes to cheater-detection mechanisms, could be subject to the same kind of detailed developmental investigation.

Recent trends in developmental biology and cognitive neuroscience recognize that the human brain and behaviour are shaped to an important extent by individual and social learning [36]. Hitherto, EP's theoretical stance led it to assume domain specificity in cognition, resulting in the neglect of opportunities to investigate to what extent human social and asocial learning are reliant upon processes that apply across domains, or the manner in which cross-domain general learning processes build on domain-specific inputs. For instance, while behavioural innovation is critical to the survival of animals living in changing and unpredictable environments, whether such innovation is channeled in a context specific manner is unclear. Innovation could instead be reliant on domain-general mechanisms expressed in complex cognition, intelligence and learning; for instance, innovation could involve learned behaviour patterns being adapted to a new domain. Available evidence suggests the latter scenario [76],[101].

Similarly, EP has engaged in a longstanding debate with advocates of cultural evolution over whether human social learning is governed by evolved content biases (e.g., choose the sugar-rich food) or by domain-general context biases (e.g., conform to the local norm). There is sufficient empirical evidence for the deployment of context biases, such as conformity or prestige bias, to render the casual dismissal of transmitted culture counterproductive [102],[103]. A broader EP could actively pursue these questions, by testing experimentally whether human social learning is dominated by content or context biases, and by investigating the factors that affect reliance on each. The finding that innovation, social learning, and other aspects of development are capable of introducing novelty into phenotype design space, thereby establishing new selective scenarios [39],[41],[48], opens up new opportunities for investigating evolutionary novelty to which social scientists can actively participate.

## Conclusions

None of the aforementioned scientific developments render evolutionary psychology unfeasible; they merely require that EP should change its daily practice. The key concepts of EP have led to a series of widely held assumptions (e.g., that human behaviour is unlikely to be adaptive in modern environments, that cognition is domain-specific, that there is a universal human nature), which with the benefit of hindsight we now know to be questionable. A modern EP would embrace a broader, more open, and multi-disciplinary theoretical framework, drawing on, rather than being isolated from, the full repertoire of knowledge and tools available in adjacent disciplines. Such a field would embrace the challenge of exploring empirically, for instance, to what extent human cognition is domain-general or domain specific, under what circumstances human behaviour is adaptive, how best to explain variation in human behaviour and cognition. The evidence from adjacent disciplines suggests that, if EP can reconsider its basic tenets, it will flourish as a scientific discipline.

## Abbreviations

AI: artificial intelligence
EEA: environment of evolutionary adaptedness
EP: Evolutionary Psychology
